# Epigenetic Therapy in Lung Cancer – Role of microRNAs

**DOI:** 10.3389/fonc.2013.00158

**Published:** 2013-06-19

**Authors:** Sacha I. Rothschild

**Affiliations:** ^1^Department Internal Medicine, Medical Oncology, University Hospital Basel, Basel, Switzerland

**Keywords:** microRNA, lung cancer, biomarker, diagnosis, prognosis, therapy

## Abstract

Lung cancer is the leading cause of cancer deaths worldwide. microRNAs (miRNAs) are a class of small non-coding RNA species that have been implicated in the control of many fundamental cellular and physiological processes such as cellular differentiation, proliferation, apoptosis, and stem cell maintenance. Some miRNAs have been categorized as “oncomiRs” as opposed to “tumor suppressor miRs.” This review focuses on the role of miRNAs in the lung cancer carcinogenesis and their potential as diagnostic, prognostic, or predictive markers.

## Introduction

Lung cancer is the most common invasive cancer and cause of cancer death worldwide (Ferlay et al., 2010). Among lung cancers, 80% are classified as non-small cell lung cancer (NSCLC) and 20% are small cell lung cancer (SCLC). While recent developments in computed tomography (CT) screening for NSCLC may lead to detection of tumors at earlier stages (Aberle et al., 2011a), currently over 70% of lung cancers are loco-regionally advanced or metastatic at the time of diagnosis. Despite advances in chemotherapy, radiotherapy, and surgery the death rate from lung cancer has remained largely unchanged. In recent years therapeutic decisions in NSCLC have been more and more based on histological and molecular characteristics. The era of molecular targeted therapy in lung cancer had its origin in 2004, when activating mutations in the epidermal growth factor receptor (EGFR) and their correlation with clinical response to EGFR tyrosine kinase inhibitors (TKIs) were discovered (Lynch et al., 2004; Paez et al., 2004; Pao et al., 2004). Other oncogenic drivers in lung adenocarcinoma include mutations of BRAF, NRAS, KRAS, MET, PIK3CA, HER2, RET, JAK2, ROS1, and ALK (Bronte et al., 2010; Garber, 2010; Janku et al., 2010; Pao and Girard, 2011). Although most of the patients with advanced NSCLC do not harbor one of the mentioned molecular alterations. Therefore, it is important to identify new markers for molecularly targeted therapy.

**Figure 1:**
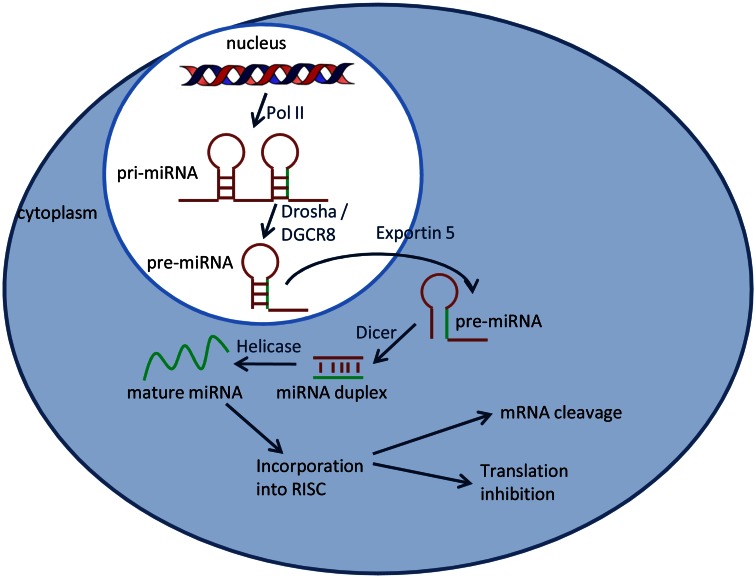
**miRNA biogenesis**. MiRNAs are transcribed by RNA polymerase II (Pol II) into long primary miRNA transcripts, which are cleaved in the nucleus by the RNase III enzyme Drosha, resulting in a hairpin precursor form called pre-miRNA. Pre-miRNA is exported from the nucleus to the cytoplasm by exportin 5 and is further processed by the enzyme Dicer which produces a transient miRNA duplex. Only one strand of the miRNA duplex (mature miRNA) is incorporated into a large protein complex called RISC (RNA-induced silencing complex).

The most studied epigenetic phenomena include posttranslational modifications in DNA and histone proteins. In the recent years the changes in expression levels of small, non-coding, single-stranded RNAs – so called microRNAs (miRNAs) – have been detected and described. miRNAs are usually 18–25 nucleotides long. Depending on the degree of homology to their 3′UTR target sequence, miRNAs induce translational repression or degradation of mRNAs (Figure 1). It is estimated that more than 1,000 miRNAs are transcribed and that 30% of the human genome is under miRNA regulation, one miRNA being able to modulate post-transcriptionally hundreds of downstream genes. In this regard, miRNAs control a wide range of biological processes including cellular differentiation, proliferation, apoptosis, and stem cell maintenance. More than half of the miRNAs genes are located in cancer-associated genomic regions or in fragile sites. Several miRNAs located in deleted regions have low expression levels in cancer tissues (Calin et al., 2004). miRNAs can act as tumor suppressors when their function loss can initiate or contribute to the malignant transformation of a normal cell. The loss of function of a miRNA could be due to several mechanisms, including genomic deletion, mutation, epigenetic silencing, and/or miRNA processing alterations (Calin et al., 2002, 2005; Saito et al., 2006; Nakamura et al., 2007). On the other hand miRNAs can act as oncogenes.

In this review the focus will be on the state-of-the-art knowledge currently available on the clinical significance of miRNAs in lung carcinogenesis and their role as diagnostic, prognostic, and predictive markers in lung cancer.

## MicroRNAs and Lung Carcinogenesis

Several studies have identified an important role of miRNAs in the regulation of the cell cycle and carcinogenesis in different organs, including the lung (Carleton et al., 2007; Chivukula and Mendell, 2008). The first evidence of miRNA deregulation in lung cancer came from the study by Volinia et al. (2006), who identified a group of miRNAs frequently aberrantly expressed in tumor tissues with respect to the normal tissue counterpart. Takahashi et al. (2009) found decreased expression of miR-107, miR-185, and let-7a, but increased expression of miR-31 in lung carcinoma tissues and cancer cell lines. Overexpression of miR-107 and miR-185 significantly reduced A549 and H1299 cell proliferation. Both miRNAs significantly increased the percentage of cancer cells in the G1 cell cycle phase. Inhibition of miR-107 increased the proliferation of A549 lung cancer cells (Cheng et al., 2005). Overexpression of let-7a, miR-107, and miR-126 suppresses lung cancer cell growth (Zhong et al., 2010).

Let-7 is an important miRNA and is highly expressed in lung tissue, as supported by *in vitro* and *in vivo* studies (Johnson et al., 2005; Yanaihara et al., 2006). The tumor-suppressive let-7 family targets well known oncogenes like Ras (Johnson et al., 2005), c-Myc (Sampson et al., 2007), and HMGA2 (Lee and Dutta, 2007) which are involved in lung cancer carcinogenesis. The let-7 family has a tumor-suppressive role in NSCLC in animal models (Kumar et al., 2008; Trang et al., 2010). It was demonstrated that 40% of lung tumors and 60% of lung cancer cell lines have low expression levels of let-7 (Takamizawa et al., 2004). In another study low let-7 expression levels were confirmed and could be correlated to high RAS expression in lung tumor models (Johnson et al., 2005, 2007). Together with further studies showing different miRNA expression profiles (Fabbri et al., 2007; Navarro et al., 2009; Raponi et al., 2009; Seike et al., 2009) seven miRNAs have frequently been found to be downregulated in NSCLC: let-7, miR-29 family, miR-30a, miR-124a, miR-126^∗^, miR-126, and miR-145 whereas six miRNAs are higher expressed in NSCLC tissue based on these studies: miR-17, miR-21, miR-106a, miR-182, miR-203, and miR-210. Own data confirmed a significant downregulation of miR-29b in lung adenocarcinoma compared to surrounding non-cancerous tissue (Rothschild et al., 2012a).

Members of the miRNA-29 family (miR-29a, miR-29b, and miR-29c) are known to be highly expressed in normal tissues and downregulated in different types of cancer, including neuroblastoma, sarcoma, glioma, high-risk chronic lymphatic leukemia (CLL), invasive breast cancer, cholangiocarcinoma, and lung cancer (Calin et al., 2005; Iorio et al., 2005; Yanaihara et al., 2006; Mott et al., 2007; Wu et al., 2009; Xu et al., 2009). The three miR-29 isoforms are arranged in two clusters: miR-29b1/miR-29a located on chromosome 7q32 and miR-29b2/miR-29c located on chromosome 1q32. miR-29a has been shown to reduce invasiveness and proliferation of human carcinoma cell lines (Muniyappa et al., 2009). In lung cancer the miR-29 family members also target DNA methyltransferases (DNMT3A and DNMT3B), and can thereby restore patterns of DNA methylation and expression of silenced tumor suppressor genes (Fabbri et al., 2007). In our own study we demonstrated direct binding or miR-29b to inhibitor of differentiation 1 (ID1) (Rothschild et al., 2012a). Anti-miR-29b enhanced ID1 mRNA and protein levels, and significantly increased lung cancer cell migration and invasion, a hallmark of the Src-ID1 pathway. miR-29b suppressed the level of ID1 and significantly reduced migration and invasion.

Hypoxia-inducible factor-1α (HIF-1α) is considered a key regulator of tumor angiogenesis (Semenza, 2010). The polycistronic cluster miR-17-92 consisting of six miRNAs (miR-17, miR-18a, miR-19a, miR-19b-1, miR-20a, miR-92a-1) is involved in embryonal lung development (Lu et al., 2007). Mice deficient for miR-17-92 die shortly after birth with lung hypoplasia and a ventricular septal defect (Ventura et al., 2008). This miRNA cluster is overexpressed in several lung cancer cell lines, preferentially in SCLC and the overexpression is associated with increased cell proliferation (Hayashita et al., 2005). The inhibition of miR-17-92 by antisense oligonucleotides can induce apoptosis in overexpressing cell lines (Matsubara et al., 2007). miR-17-92 targets HIF-1α which might be the key mechanism of action in lung cancer cell proliferation (Taguchi et al., 2008). Moreover, miR-519c binds to the HIF-1α 3′UTR and thereby reduces tumor angiogenesis (Cha et al., 2010).

Retinoblastoma 1 (Rb1) was the first identified tumor suppressor. Overexpression of miR-192 inhibited cell proliferation in A549, H460, and 95D lung cancer cells by decreasing Rb1 mRNA and protein expression (Feng et al., 2011). The same effect was demonstrated in a nude mouse model.

Epidermal growth factor receptor signaling has been shown to play an important role in cell migration and invasion (Prenzel et al., 2001). miR-125a-5p is regulating several downstream genes involved in EGFR signaling (Wang et al., 2009). Inhibition of miR-125a-5p significantly enhanced cell migration and invasion, suggesting that miR-125a-5p is negatively correlated with lung cancer invasion and metastasis.

Further miRNAs that have been described to have tumor-suppressive function in lung cancer are miR-93 (Du et al., 2009), miR-98 (Du et al., 2009), miR-101 (Zhang et al., 2011), miR-182 (Sun et al., 2010), miR-197 (Du et al., 2009), miR-212 (Incoronato et al., 2010), miR-451 (Wang et al., 2011).

Recently, it has been shown that miRNAs are also present in body fluids, shuttled by so called exosomes, which are secreted both by normal and tumor cells. Two miRNAs (miR-23 and miR-225) are expressed at higher levels in the circulating exosomes of NSCLC patients compared to healthy donors (Chen et al., 2008). There seems to be a correlation between the expression of miRNAs in tumor tissues and in the blood suggesting that the tumor releases circulating miRNAs inside exosome particles (Rabinowits et al., 2009). A recent publication identified specific miRNAs (e.g., miR-21 and miR-29a) in exosome of lung cancer patients able to bind and activate Toll-like receptors (TLR) in cancer surrounding immune cells (Fabbri et al., 2012). By activating TLRs, these miRNAs trigger the NF-kappaB pathway which ultimately leads to an increased production of interleukin-6 (IL-6) and tumor necrosis factor-alpha (TNF-alpha) by the immune cells, leading to increased tumor growth and metastatic potential. This study describes a novel mechanism of action of miRNAs and support the role of cancer-released exosomes in lung cancer carcinogenesis.

In general, the current literature suggests that miRNAs are involved in lung cancer carcinogenesis by different mechanisms. They might act directly by targeting oncogenes or tumor suppressor genes and regulate their expression or by influencing their epigenetic regulation within the tumor. As a novel mechanism, miRNAs also affect lung carcinogenesis by triggering a TLR-mediated oncogenic inflammatory response.

## MicroRNA as Diagnostic Markers of Lung Cancer

Since there is no validated population-based screening procedure available, most patients with lung cancer are diagnosed at advanced stages. Recently a large trial showed that low-dose helical CT screening in an older, high-risk population reduced lung cancer mortality by 20% (Aberle et al., 2011a,b). However there is a high number of false positive findings in CT scans (Aberle et al., 2011a; Barba et al., 2011). Therefore, development of a reliable and non-invasive confirmatory test would reduce overdiagnosis. Interestingly, miRNA expression levels show apparent differences following exposure to cigarette smoking (Izzotti et al., 2009). Therefore changes in miRNA expression might be a reliable tool for cancer early detection in smokers. Xie et al. (2010) found that miR-21 expression in the sputum specimens was significantly higher in cancer patients compared with healthy controls. Further studies have shown that increased miR-21 expression resulted in 70% sensitivity and 100% specificity in the diagnosis of lung cancer by sputum cytology (Wang et al., 2012). Therefore, investigating miRNA expression levels in the sputum might be a potential non-invasive approach for early lung cancer detection.

Yanaihara et al. (2006) studied miRNA expression profiles in lung cancer tissues and normal adjacent tissue. They identified a unique miRNA profile allowing discriminating lung cancers from non-cancerous lung tissues. Furthermore they describe a signature that distinguishes histological subtypes of NSCLC. Some of the miRNAs (e.g., miR-21, miR-155, and members of the miR-17-92 cluster) were common to the two studies, and have been found to be upregulated in other types of cancer. Shen et al. (2011) identified 12 miRNAs aberrantly expressed in early-stage NSCLC. Four of them (miR-21, miR-126, miR-210, and miR-486-5p) yielded 86% sensitivity and 97% specificity in distinguishing NSCLC patients from healthy controls. Furthermore, these miRNAs had higher sensitivity (92%) in diagnosing lung adenocarcinoma compared with squamous cell carcinomas (82%) (*p* < 0.05). In a recent study miRNA expression in tumor and non-cancerous tissue pairs collected from stage I-III lung squamous cell carcinoma patients treated with neoadjuvant chemotherapy or radiotherapy were analyzed (Tan et al., 2011). Five miRNAs (miR-30a, miR-140-3p, miR-182, miR-210, and miR-486-5p) were identified to distinguish invasive lung cancer from non-cancerous lung tissue. The classifier had an accuracy of 94.1% in a training cohort (34 patients) and 96.2% in a test cohort (26 patients). Yu et al. (2010) found that the combined overexpression of miR-21, miR-200b, miR-375, and miR-486 in surgical tissues and sputum were biomarkers in the prediction of lung adenocarcinoma from normal controls with 81% sensitivity and 92% specificity. The results have been validated in 64 lung cancer patients and 58 cancer-free participants. The detection of miR-205, miR-210, and miR-708 in sputum was highly sensitive (73%) and specific (96%) in diagnosing lung cancer (Xing et al., 2010).

Compared with normal lung tissues, the top 10 deregulated miRNAs in lung tumors that were appropriate for discriminating CT-detected lung cancer from normal lung tissue were let-7, miR-21, miR-200b, miR-210, miR-219-1, miR-324, which were all upregulated, and miR-30a, miR-126, miR-451, and miR-486, which were all downregulated (Boeri et al., 2011).

The histopathological subclassification of NSCLC into the two large groups adenocarcinoma and squamous cell carcinoma has gained interest during the last years because adenocarcinoma histology in stage IV NSCLC is predictive for treatment with pemetrexed (Scagliotti et al., 2009). Two miRNAs (miR-21, miR-205) were reported to accurately distinguish adenocarcinoma from squamous cell carcinoma subtype (Lebanony et al., 2009).

Moreover, different miRNA expression profiles were also described between primary lung tumors and metastases. Overexpression of miR-182 was found in primary lung cancers, whereas miR-126 was highly expressed in metastatic tumors (Barshack et al., 2010).

In conclusion, these results suggest that miRNA signatures can help to differentiate lung cancer tissue from non-cancerous lung tissue. Furthermore, there are specific miRNAs associated with early lung cancer development which could be measured in sputum specimens and might support early lung cancer diagnosis. Other miRNAs can help to distinguish between histopathological subtypes of NSCLC or between lung primary tumors and metastases.

## MicroRNAs as Prognostic Biomarkers of Lung Cancer

At least two studies demonstrated the prognostic role of let-7 in patients with NSCLC. Takamizawa et al. (2004) investigated 159 patients with stage I-III NSCLC and showed that low expression of let-7 was associated with a shorter postoperative survival. This finding was confirmed by Yanaihara et al. (2006) showing that overexpression of the precursor of miR-155 and the downregulation of let-7a-2 correlated with poor survival in NSCLC patients. This result was confirmed in a further study (Chin et al., 2008). In a study by Yu et al. (2008) let-7a was part of a signature of five miRNAs (let-7a, miR-137, miR-182^∗^, miR-221, miR-372) that were prognostic predictors. Reduced expression of miR-146b was found to be associated with a reduced overall survival in patients undergoing radical surgery for squamous cell carcinoma of the lung (Raponi et al., 2009). Another study in stage I-III squamous cell carcinoma showed a negative prognostic impact of high miR-31 expression (Tan et al., 2011). In male smokers with stage I-IIIA squamous cell of the lung, Landi et al. (2010) identified a signature of five downregulated miRNAs (let-7e, miR-25, miR-34a, miR-34c-5p, miR-191) predicting for poor outcome. miR-328 might be a marker for patients at higher risk for brain metastases as this miRNA has been found to be differentially expressed in patients with and without brain metastases of NSCLC (Arora et al., 2011). Lower expression of miR-34a in tumor tissues was associated with a high recurrent risk (Gallardo et al., 2009). High miR-21 and low miR-181 expression levels predicted poor survival independent of TNM staging (Gao et al., 2010). In another study overexpression of miR-429 was associated with shorter disease-free survival and downregulation of miR-486-5p in plasma predicted for poor outcome (Donnem et al., 2011).

The role of adjuvant chemotherapy in surgically resected stage I NSCLC is controversial (Besse and Le Chevalier, 2012). Reliable biomarkers predicting the risk of relapse are needed to identify patients most likely to benefit from adjuvant chemotherapy. Patnaik et al. (2010) demonstrated that miRNAs can predict lung cancer recurrence with an accuracy of 83%. Lu et al. (2012) reported on 527 stage I NSCLC patients investigated for miRNA expression levels. Two miRNA signatures were associated with relapse-free survival. The first contained 34 miRNAs derived from 357 patients whereas the second was based on 27 miRNAs from adenocarcinoma histologies. Both signatures were validated in an independent data set with 170 stage I NSCLC patients.

In the largest cohort of NSCLC patients investigating the prognostic and predictive value of miRNA signatures 639 patients with radically resected stage I-III NSCLC randomized to adjuvant cisplatin-based chemotherapy or follow-up [IALT trial (Arriagada et al., 2004)] were included (Voortman et al., 2010). miRNA expression levels in the tumor tissue were analyzed using quantitative real-time PCR (qRT-PCR). Only low expression of miR-21 was associated with shorter survival time. None of the investigated miRNAs nor any of the tested signatures was of predictive value for adjuvant chemotherapy. Hu et al. (2010) described a signature of circulating miRNAs (miR-1, miR-30d, miR-486, miR-499) associated with the outcome in 303 surgically resected stage I-IIIA NSCLC patients treated with adjuvant chemotherapy.

In our own study, we extracted total RNA from tumors and matched non-cancerous lung tissue from 23 cases with a pathological diagnosis of lung adenocarcinoma (Rothschild et al., 2012a). miRNA expression levels were determined using qRT-PCR. In 19 (83%) of the cases, miR-29b was downregulated in tumor compared with matched lung. Using the median tumor level as cut-off value, tumor miR-29b expression significantly correlated with event-free (*p* = 0.003) and overall survival (*p* = 0.039). In another study we extracted total RNA from 18 tissue samples of lung adenocarcinoma and also from non-cancerous tissue from the same patients (Rothschild et al., 2012b). In 15/18 (83%) of analyzed adenocarcinoma samples, miR-381 was downregulated in tumor compared with normal lung tissue. Overall miR-381 expression was significantly lower in adenocarcinomas than in matched normal lung tissue (Mann–Whitney *U* test, *p* = 0.003). Using the median tumor level as cut-off, tumor miR-381 expression significantly correlated with event-free survival (*p* = 0.003) and overall survival (*p* = 0.02).

In conclusion, several miRNAs show tissue specific expression levels and can be correlated with patient outcome.

## MicroRNA as Predictive Markers in Lung Cancer

As discussed in the section on miRNA as prognostic markers, the largest clinical trial evaluating the role of miRNA signatures in NSCLC did not show any of the examined miRNAs to be of predictive value (Voortman et al., 2010). Nevertheless, several preclinical studies as well as miRNA expression analyses from clinical trials showed that certain miRNAs might be of predictive value and do influence the sensitivity of lung cancer cells to chemotherapeutic or targeted agents as well as to irradiation.

In our own study we raised the hypothesis that miR-29b is a potential predictive marker for SRC TKIs due to the fact that inhibition of miR-29b diminished the effects of SRC tyrosine kinase inhibition (Rothschild et al., 2012a).

miR-17-92, miR-221, and miR-222 have been shown to sensitize lung cancer cells to cytotoxic agents (Hayashita et al., 2005; Matsubara et al., 2007; Garofalo et al., 2008). Galluzzi et al. (2010) demonstrated that miR-181a and miR-630 modulate the sensitivity of lung cancer cell lines toward cis-diamminedichloroplatinum (cis-DDP). pre-miR-630 diminished the sensitivity of A549 cells in response to DDP by inducing cancer cell arrest in G0-G1 and increasing the cell cycle inhibitor p27^*kip*1^. Ectopic expression of miR-451 on the other hand sensitized A549 cells to DDP possibly by increasing DDP-induced apoptosis (Bian et al., 2011).

The description of oncogenic mutations (e.g., EGFR mutations) and the introduction of new therapeutics directly targeting these molecular changes (e.g., EGFR TKIs) have changed the therapeutic strategies in lung cancer treatment. Therefore, higher interest in understanding sensitivity and resistance mechanisms toward these new therapeutic approaches is a consequence of this development. Higher expression of miR-126 enhanced cytotoxicity induced by gefitinib in lung cancer cells (Zhong et al., 2010). EGFR is directly targeted by miR-128b and miR-128b loss-of-heterozygosity (LOH), which is frequently found in NSCLC, is a direct regulator of EGFR, correlates with clinical response and survival following gefitinib therapy (Weiss et al., 2008). Also, miR-21 was shown to enhance the therapeutic effects of EGFR TKIs in lung cancer cell lines (Seike et al., 2009). miR-7 has been described to target EGFR, AKT, and ERK and therefore suppressing EGFR pathway signaling (Webster et al., 2009). Ectopic expression of miR-7 was able to overcome EGFR TKI resistance in lung cancer cell lines (Rai et al., 2011). Another study described that restoration of tumor suppressor miR-145 inhibits cancer cell growth in EGFR mutant lung adenocarcinoma (Cho et al., 2009).

TRAIL (Apo2L/tumor necrosis factor-related apoptosis-inducing ligand) is a member of the TNF family that can induce apoptosis in several types of cancer, including lung cancer (Schaefer et al., 2007). miR-221 and miR-222 silence PTEN and TIMP3 tumor suppressors and therefore lead to resistance of lung cancer cells toward TRAIL (Garofalo et al., 2009).

The role of miRNA expression related to irradiation has been investigated by several groups. A549 cells, which contain lower let-7 levels and higher activated RAS had significant changes in miRNAs as early as 2 h after irradiation (Weidhaas et al., 2007). Inhibition of let-7b caused significant protection from radiation (Galluzzi et al., 2010). On the other hand, overexpression of let-7g protected A549 cells from radiation, whereas inhibition of let-7g increased radiosensitization in lung cancer cells (Galluzzi et al., 2010).

In conclusion, despite a large negative study on the predictive role of miRNAs signatures in NSCLC further studies are warranted on the basis of several preclinical data. As shown in various studies miRNA can modulate the response of lung cancer cells to EGFR TKIs by interacting with the respective signaling pathway. Confirmation of their predictive role in larger clinical trials is needed.

## Conclusion

Since their discovery in the 1990s miRNAs have increasingly been recognized as key player in carcinogenesis and cancer progression, but also as potential diagnostic markers and biomarkers. In this overview more insights in the involvement of miRNAs in lung carcinogenesis as well as in their role as diagnostic, prognostic, and predictive biomarkers have been summarized. The current evidence on the prognostic and predictive role of miRNAs is at least partially inconsistent. Therefore, prospective trials evaluating the role of miRNAs described to have prognostic or predictive impact in small retrospective analysis should be planned. For this, standardized protocols for the isolation and the analysis of expression levels of miRNAs are needed.

For the future, miRNAs from body fluids, especially the blood plasma might be the center of attention. As discussed, numerous studies have identified aberrant miRNA expression profiles in lung cancer. Therefore, miRNAs can potentially be used as biomarkers in the diagnosis and classification of lung cancer. Moreover, miRNA expression levels in blood plasma may be used as a non-invasive confirmatory screening test complementary to the low-dose helical CT screening in the near future.

Another interesting research field is the use of miRNAs as therapeutic agents. The miRNA replacement or the anti-miRNAs will possibly become a new strategy for lung cancer therapy. At the moment several challenges have to be resolved before miRNAs could be investigated in clinical trials. One of the main problems is to find a suitable non-toxic delivery system capable of selectively transporting miRNA-based therapeutics to the tumor site. As discussed in the introduction, one single miRNA is able to target more than 100 mRNAs. It is therefore highly questionable if miRNAs as therapeutic tools could be of sufficient specificity.

In conclusion, current knowledge on miRNAs in lung cancer has extended our understanding of lung cancer carcinogenesis and provides health care professionals with a new and innovative cancer biomarker. Beside preclinical research, there are several clinical trials evaluating the role of miRNAs as prognostic and/or predictive biomarkers ongoing.

## Conflict of Interest Statement

The authors declare that the research was conducted in the absence of any commercial or financial relationships that could be construed as a potential conflict of interest.
